# Some Diagnostic Points of Mental Diseases and Why the Commitment Proceedings of the Insane of Georgia Should Be Entirely Revised

**Published:** 1910-10

**Authors:** J. Cheston King

**Affiliations:** Atlanta, Ga.


### Journal-Record of Medicine

Successor to Atlanta Medical and Surgical Journal, Established 1855, and Southern Medical Record, Established 1870.

OWNED BY THE ATLANTA MEDICAL JOURNAL CO.

Published Monthly

*Official Organ Fulton County Medical Society, State Examining Board, Presbyterian Hospital, Atlanta, Birmingham and Atlantic Railroad Surgeons' Association, Chattahoochee Valley Medical and Surgical Association, Etc.*

EDGAR G. BALLENGER., M. D., Editor.
BERNARD WOLFF, M. D., Supervising Editor.
A. W. STIRLING, M. D., C. M., D. P. H., J. S. HURT, B. Ph., M. D.
GEO. M. NILES, M. D., W. J. LOVE, M. D., (Ala.); Associate Editors.
E. W. ALLEN, Business Manager.

COLLABORATORS

Dr. W. F. WESTMORLAND, General Surgery.
F. W. McRAE, M. D., Abdominal Surgery.
H. F. HARRIS, M. D., Pathology and Bacteriology.
E. B. BLOCK, M. D., Diseases of the Nervous System.
MICHAEL HOKE, M. D., Orthopedic Surgery.
CYRUS W. STRICKLER, M. D., Legal Medicine and Medical Legislation.
E. C. DAVIS, A. B., M. D., Obstetrics.
E. G. JONES, A. B., M. D., Gynecology.
R. T. DORSEY, Jr., B. S. M. D., Medicine.
L. M. GAINES, A. B., M. D., Internal Medicine.
J. N. LeCONTE, M. D., Disease of the Stomach and Intestines.
L. B. CLARKE, M. D., Pediatrics.
EDGAR PAULIN, M. D., Opsonic Medicine.
THEODORE TOEPEL, M. D., Mechano Therapy.
R. R. DALY, M. D., Medical Society.
A. W. STIRLING, M. D., etc., Diseases of the Eye, Ear, Nose and Throat.
BERNARD WOLFF, M. D., Diseases of the Skin.
E. G. BALLENGER, M. D., Diseases of the Genito-Urinary Organs.

VOL. LVI. OCTOBER, 1910 No. 7.

### SOME DIAGNOSTIC POINTS OF MENTAL DISEASES AND WHY THE COMMITMENT PROCEEDINGS OF THE INSANE OF GEORGIA SHOULD BE ENTIRELY REVISED.

BY J. CHESTON KING, A. B., M. D., ATLANTA, GA.

Before bringing out any of the cardinal points of my subject, I wish to impress upon this medical body that mental disease is the result of disease of the brain, and is not a mere immaterial disorder of the intellect. The brain is an organ through which mental phenomena is manifested, and therefore, it is impossible to conceive of an insane mind in a healthy brain.

Twenty-three centuries ago the learned physicians of Greece and Rome had emancipated themselves by scientific induction from brutal superstitions, and they clearly and boldly taught that *insanity was brain disease* with prominent psychical symptoms; that there are mental and bodily causes; that medical and diatetic means of cure should be employed.

It was Hypocrates, 460 B. C., who propounded certain truths which formed the basis of the science of psychiatry. He demonstrated that the brain was the organ of the mind, and that it was subject to physical laws and diseases like other organs, and that insanity followed abnormal conditions of the brain.

Before this time the civil and social status of the insane and their treatment were determined by religious and superstitious hypotheses; they were regarded as afflicted by the gods or possessed by demons. Do you know that to even this day and time, there is among the laity some ramanants of the middle ages? They absolutely act towards and speak of the mentally unbalanced with hushed breath and mortification.

But the middle ages at last gave way to the philosophic phase of medical science of the seventeenth and eighteenth century after the appearance of Kepler and Galileo, after Descartes had founded modern philosophy, after Bacon had aroused the thinking world by his inductive method in the study of nature, and after Harvey had published his researches on the circulation of the blood.

It was in the eighteenth century through the labors of Jacobi, Vering, and Fredrich, all of Germany, that there was a systematic study of the Somatic symptoms and to the doctrines that physical disease was the cause of insanity.

The Somatic School established a belief in the cure of insanity, a point of great importance with reference to the general amelioration of the condition of the insane, who had ordinarily been dealt with as beyond hope of cure.

So from these rapid advances of the human mind, we can but truly feel as the author, who said: "That knowlege is not a couch whereon to rest a seaching and restless spirit, or a terrace for a wandering and variable mind to walk up and down, with a fair prospect, or a tower of the State for a proud mind to raise itself upon, or a fort, or commanding ground for strife and contention, or a shop of profit and sale; but a rich storehouse for the glory of the Creator and the relief of man's estate."

Before giving out some diagnostic points of mental diseases, a classification is necessary. Each part of the brain has its special function, and to a certain extent is independent of each other.

One is essential to vital processes, hence its destruction causes death; another part presides over the various movements of the body, hence paralysis of motion is the result of destruction of any portion of this area; a third part enables the mind to appreciate touch, temperature and pain; a fourth presides over sight: and in the small way smell, hearing, and taste are governed by distinct portions of the brain.

In our contact with mental diesases, four leading classes stand out prominently before us, viz.: Amentia, Dementia, Mania, and Melancholia.

Amentia. Under this heading there are two forms: (1) Idiocy and (2) imbecility.

By describing idiocy we mean a congenital malady, and the idiot is one, who by reason of his perpetual infirmity, is *non compos mentis*. The vitality of the general system is less than normal, the palate is vaulted and narrow, the teeth misshapen, wrongly placed and prone to decay, the patient is dwarfish, the head unsymmetrical, and sometimes the commissures are atrophied.

IMBECILITY. This term is distinguished from *Idiotism* in that the mental defect is manifested in the first years of childhood, and does not reveal itself as early as idiotism. True, the fainter shades of imbecility pass into the lighter tints of idiocy. But the possession by the imbecile of the faculty of speech as distinguished from the parrot-like utterances of the few words which the idiot can learn, is one of the best lines of demarcation.

This class of children, as a rule, are unruly, vicious and cruel. They are intellectually as well as morally deficient, and cannot control their emotions or passions.

There is a class of imbeciles who exhibit intellectual deficiency without offending against morality, and still another class, who combine the highest intellectual endowment with utter incapacity in the conduct of life—so we have the intellectual, moral, and general imbecile.

The form of imbecility which is most common and most important in a medico-legal point of view is that which affects the intellect, the morals and the conduct of life.

Persons who exhibit this three-fold deficiency, profit by education so as to form and express ideas, to read, write, and count, to become musicians, draughtsmen or mechanics. They may even attain proficiency in some branch of knowledge; but they do not profit by opportunities afforded them. They present great varieties of character. Some are fickle and changeable and incapable of fixing their attention, others methodical and persevering. Some are fit for the coarest labors, while others duly assisted and guided are equal to the conduct of business and management of business, but are unequal in emergencies and unable to sustain close conversation or argument. They have a very imperfect idea of the laws of society, morality, courts and trials; and though they may have an idea of property, they have no conception of the consequences of theft.

Some writer has said: "These beings of limited capacity furnish to the courts of justice, to prisons and scaffolds, more subjects than is generally supposed."

The moral imbecile remains at large, because the intellect being unaffected, they have no distinct delusion; and as weakness of intellect is a necessary ingredient in the legal idea of imbecility, the attempt to prove such persons of insane mind in a court of law necessarily fails.

That absence of moral sense and corresponding want of self-control, which is the essence of their mental malady, can be proved only by the history of their daily life; a history often hard to explain and generally studiously withheld. There is one class of imbeciles that should not be overlooked. They are very apt and really precocious in the first few years at school, but at puberty their mental capacities retrograde, and what they have accomplished in the past vanishes, and mental development closes. Now this occurs suddenly.

The question of responsibility for such acts as arson, and murder can only be answered by weighing well all the circumstances of the act, and the whole life and character of the accused, and ascertaining the motive by which the act was instigated. It is in imbeciles more than in other persons of insane minds that the test of the knowledge of right and wrong utterly breaks down. So from what I have said on idiotism and imbecility, the boundry line between the two is evanescent. The imbecile patient is capable of pursuing some calling, and the idiot simply occupies space and has no voice in the body politic.

DEMENTIA is a functional psychosis, manifested by paralysis of the mental faculties.

It is distinguished from the two forms of amentia just described, in that it supervenes slowly or suddenly in the mind already fully developed, and in childhood, manhood, or old age. It differs from mania, for it consists in exhaustion and torpor of the faculties, not in violent and sustained excitement.

We recognize an acute and chronic form of it. The first is rare and consists of a profound melancholy; the second is common and characterized by incoherence, but said incoherence is different from the incoherence of mania, for it is void of excitement. There is also a Senile Dementia. Dementia has its stages of forgetfulness, irrationality, incomprehension and inappetency We find in this class of patient a want of memory, then loss of reasoning, lack of ability to comprehend, the pulse is retarded, and the temperature is subnormal, and later there is an abolition of the common instructor of volition.

In acute dementia, caused from sudden mental shocks, the mind seems fixed and arrested in sad abstraction on the event which occasioned it.

The chronic form of Dementia is often brought on by some slow acting cause, such as prolonged grief or anxiety, sometimes it follows fever, apoplexy or paralysis.

It has been ascertained that inhalation of a poisonous vapor like mercuric methide can induce in a perfectly healthy man a state of brain passing gradually into the most hopeless dementia.

SENILE DEMENTIA arises from causes acting slowly and gradually. The first symptom is impaired memory of recent events, with dullness of perception and apprehension, and inability to fix the attention or follow any train of thought. The things heard five minutes since are forgotten. Memory, reason, and power of attention are entirely lost, but the muscular force remains intact.

In the acute forms of this mental trouble, under proper environment and treatment, about 80 per cent. of the cases recover. It lasts from a few days to several months.

MANIA. This term includes all the forms of unsoundness that are characterized by undue excitement, while in melancholia there is hyperaesthesia of the feelings, while in paranoia, the primary element is the disturbance of the laws of association.

The delusions of the paranoiac and the melancholiac are practically the same. The line of distinction lies in the fact that the paranoiac believes that his persecutions are unwarranted, that men are enemies of his—without cause. He defends himself and defies his persecutors. The melancholiac feels that God, through his aggressors has justly punished him for his transgressions.

General mania affects the intellect, the emotions, and the passions, and throws the whole mind into a state of mingled excitement and confusion.

Mania, no matter what form it is, unless it be the immediate consequences of injuries, moral shocks, intoxication, poisoning, or acute diseases, is commonly preceded by important bodily and mental changes, which occupy a variable period from a few days to fifteen or twenty years.

If the period of incubation is short, the disease shows itself at the end of some hours or days of anxiety, uneasiness, and sadness, by headache, sleeplessness and excitement. The patient begins to cry and sing, becomes wild and agitated, and seems like a person intoxicated. Now, when the period is extended, the disease generally begins with a consciousness of some disorder of the mind, characterized by odd motions, unusual inclinations, and changing affections. The patient is vexed at the change and tries to conceal it; continues his occupation; and like a man in the first stages of intoxication, makes every effort to conceal it. Meanwhile his health gives way. He suffers from insomnia, he loses flesh. His habits, his tastes and his affections become entirely reversed. If his nature was open and candid, he becomes suspicious and jealous. His entire nature changes to the extreme. After the period of incubation has passed, the disease fully established, the patient has faith in his delusions, no longer conceals his thoughts, but openly proclaims them. When opposed he uses violent measures. He sustains a long time without sleep. Then we have that form of mania known as partial sensory types, in which there are illusions and hallucinations.

Also monomaniacs are of a class of cases of general intellectual mania, in which the senses are the sport of illusions.

Hoffbauer has maintained: "That mania may exist uncomplicated with mental delusion; it is, in fact, only a kind of mental exaltation, a state in which the reason has lost its empire over the passions and the actions by which they are manifested to such an extent that the individual can neither repress the former, nor abstain from the latter. It does not follow that he may not be in possession of his senses and even of his usual intelligence, since in order to resist the impulses of his passions, it is not sufficient that reason should impart its counsels, he must have the power to obey."

We now come to our last classification, viz.:

MELANCHOLIA which is a psychosis, characterized by the most exaggerated feelings of depression and gloom.

The patient is always sad and full of the most anxious forebodings, with a distaste for all his former interests and pursuits, but without definite delusions. He feels that he has been damned, that he has committed the unpardonable sin—and to use the exact expression of one of my patients—that he is ten thousand times the meanest man in hell.

His misery appears so unendurable that if the opportunity is offered, he resorts to suicide. Marked impairment of health and nutrition is generally associated with it. There is loss of appetite, feeble circulation, emaciation, obstinate constipation, furred tongue, a dry, harsh skin—sleeplessness is a usual symptom. Melancholia is a disease of middle life, and is generally attributed to grief, anxiety, over-work. Hereditary predisposition is sometimes an important factor.

Melancholia, especially of the hypondriac type, often passes into dementia.

What has been said is but a very brief outline of some condensed diagnostic points of our modified classification of mental diseases.

For a few moments we will now dwell on the commitment proceedings of Georgia in reference to its insane and endeavor to show why the law governing same is not in advance of the times. Since the civil and social status of the insane and their treatment is no longer determined by religious and superstitious hypotheses, and they are not regarded as possessed by demons, then should our laws governing their entrance into State institutions be most humane, prompt and effective,—and the trial by jury of these unfortunates be relegated to the dark ages.

I claim that to subject the insane to a trial by jury has a vetsige of cruelty, and there is no more sense in submitting the sanity or insanity to a jury of laymen, than there is in presenting before said body the existence of pellagra. We should have a law that would act speedily to the insane because of their illusions, rather than a law as we have that places the criminal in the same category as the unfortunate insane.

Insanity is necessarily a medical proposition, and whether a person be tried by jury or a commission, the case is based on medical opinion, and too much emphasis cannot be placed on the fact that incipient and acute cases of insanity should have the earliest scientific treatment.

Now, how do our Georgia laws operate in reference to its insane? Perhaps a young mother develops mania—after childbirth. She is tried before a jury of six laymen and one physician, in the same manner as a thief or murderer. After a verdict is reached several days must elapse before she can be removed to the state sanitarium for rest and medical attention. If she is poor and her people have not the income to place her in a private institution, and she cannot be cared for at home—where is she placed in the intervening time? In jail—whose companions are murderers, abandoned women, rats and vermin. When there is an epidemic of contagious or malignant diseases in our State, is it necessary to have a commitment by jury before the persons so afflicted can be placed in pest houses? No—yet does not the same apply to people who are insane? Does not the community demand protection at once?

Commitment by jury is an antiquated method and is a great hardship to the patient, and most humiliating to the relatives—and is but a remnant on our statue books of Egyptian darkness. What do the laws of the various states of the Union say?

Alabama.—Judge of probate may act with or without verdict of the jury, at his discretion.

Arizona.—Probate judge may act.

Arkansas.—Probate judge or county judge may act.

Colorado.—County court or judge, and jury if the insane person so select.

Connecticut.—Judge of superior court may act.

California.—Judge or jury (if demanded) upon proper testimony.

Delaware.—Written order of chancellor or State trustees.

Florida.—Circuit judge or county judge.

Georgia.—Petition to the ordinary, who must act upon the verdict of seven men.

Illinois.—Petition to the judge of the county court, and a jury of six persons, one of whom shall be a physician.

Indiana.—Two justices of the peace and a respectable practicing physician.

Iowa.—Commissioners of insanity receive applications, make inquiry into the patient's condition, hear evidence, etc., with power to commit. (See also laws of Dakota.) May appeal to district court from findings of commissioners.

Idaho.—Any judge of court of record may act.

Kansas.—Probate judge or jury of four men, one of whom shall be a physician.

Kentucky.—Circuit court judge and jury required.

Louisiana.—Judge of the district or parish court may act.

Maine.—Municipal officers of the town constitute board of examiners. Commitment on the certificate of at least two respectable physicians, based upon view, inquiry, and personal examination, etc.

Massachusetts.—Judge of supreme court or superior court or a judge of probate court may commit any insane person who is a proper subject for treatment or custody. No commitment without the certificate of two physicians, each of whom to be a graduate, etc. Violent persons may be received at hospital without warrant of commitment for not exceeding five days without an order of the judge, as provided.

Michigan.—The judge of probate or jury of twelve men.

Minnesota.—Judge of probate may act, assisted by two persons, one of whom shall be a duly qualified physician, and the three shall constitute a jury, etc.

Missouri.—Probate court shall cause inquiry and decision by jury to be made.

Montana.—A district judge acts. Must subpœna two physicians to make examination and testify and sign certificate.

New York.—Certificate of two physicians under oath, setting forth the insanity of such person within five days from day of confinement. Certificate must be approved by a judge or justice of a court of record of the county or district, etc. Judge may institute inquiry and make proofs as to lunacy before approving or disapproving such certificate. Judge may, in his discretion, call a jury to determine the question of lunacy.

New Jersey.—Order of court or common pleas court judge authorized to send patients, etc., and request, etc., having been first lodged with the superintendent of the hospital asylum upon certificate of two physicians.

New Hampshire.—Order of court or the judge of probate on certificate of two respectable physicians, etc., and such certificate shall be accompanied by a certificate of the judge of the supreme court or a court of probate, a mayor, or a chairman or a selectman testifying to the truthfulness of the signatures of the signee.

Nebraska.—Commissioners may act. Must have testimony of one physician.

Nevada.—District judge and two physicians.

New Mexico.—Commission and jury, as the circumstances may seem to require.

North Carolina.—Clerk of the superior court acts on testimony of at least one physician.

Maryland.—Circuit court of the county, etc., shall cause a jury of twelve to be empaneled and pass upon the question, etc.

Ohio.—Probate judge on certificate of medical witnesses.

Oregon.—County judge may act. Testimony of one or more physicians.

Pennsylvania.—Two or more reputable physicians under a personal examination made within one week of the date of their certificate, and the certificate to be duly acknowledged and sworn to or affirmed before a jury or magistrate of the county that such person has been examined, who shall certify to the genuineness of the signatures, etc., and the standing and good repute of all of the signers.

So it is seen that only six states of our Union require commitment by jury. In fact, most of the European countries, in dealing with insanity cases, have done away with the inquisition by jury. Our Georgia law in this respect is just about one hundred years behind the pace of progress.

This association should use its influence in having enacted a new lunacy law, eliminating the necessity of a jury trial. There should be a regular form for the commitment of a patient made out by two nearest relatives—setting forth why they believe the patient is of unsound mind; an affidavit should then be made by two competent physicians, certifying that they have made an examination of the mental condition of the person, and that in their judgment, said person is of unsound mind, and is a fit subject for treatment at the state sanitarium.

The judge of the superior court or the ordinary's court issue thereupon an order committing at once the patient to the state institution.

And on this line of state legislation, without going into the duties, powers, etc., there should be enacted a law for the appointment of a State Commission of Lunacy, with compensation fixed by the state, and every civil and criminal case into which is injected the plea of insanity, should be examined by this commission and their findings announced.

Then we shall have arrived at the recognition that insanity is a physical disease, and that judges need expert medical advice instead of the opinion of a jury of laymen.

When we shall have accomplished this, then we shall have passed out of the darkening storms of primitive civilization, and emerged into noontide splendor of moral grandeur and usefulness, upon which all the people under the sun may point to medical research with pride and hope.

---

The above article was read before the Fulton County Medical Society, on May 5, 1910, and Drs. Baird, Clark, Daly and Kime discussed at length the paper. On motion of Dr. Kime, it was unanimously endorsed, and a committee of the following members, namely, King, Kime and Daly were appointed by the Chair to meet with the standing law committee and formulate a bill in accordance with the views expressed in the paper read by Dr. King, and have same presented to the legislature for adoption.

Some two weeks later in May, the author of the article was invited to read same before the Woman's Confederated Clubs. The time being limited for a discussion of this paper, proposing to change the present Commitment laws of Georgia, Dr. King was invited to present at the afternoon session a synopsis of his views. This was done, and a full discussion of the paper followed by the members. After which, a motion was made and carried that the Woman's Confederated Clubs are in sympathy with the views expressed by Dr. King, and endorse the changes he proposes in the Commitment Proceedings of the State of Georgia.

The editor understands that the bill has been formulated, and the Hon. Hooper Alexander, will present same at the next legislature.

